# Epidemiology and Economic Outcomes Associated with Timely versus Delayed Receipt of Appropriate Antibiotic Therapy among US Patients Hospitalized for Native Septic Arthritis: A Retrospective Cohort Study

**DOI:** 10.3390/antibiotics11121732

**Published:** 2022-12-01

**Authors:** Joan-Miquel Balada-Llasat, Nicole Stamas, Tom Vincent, Tristan T. Timbrook, Cynthia Saiontz-Martinez, Rachael B. Hemmert, Ariel Berger

**Affiliations:** 1 Department of Pathology, The Ohio State University, Columbus, OH 43203, USA; 2 Evidera, Bethesda, MD 20814, USA; 3 BioMérieux, Salt Lake City, UT 84104, USA

**Keywords:** arthritis, antibiotic, delayed, health care utilization, cost measures

## Abstract

Timely administration of appropriate antibiotic therapy is associated with better patient outcomes and lower costs of care compared to delayed appropriate therapy, yet initial treatment is often empiric since causal pathogens are typically unknown upon presentation. The challenge for clinicians is balancing selection of adequate coverage treatment regimens, adherence to antimicrobial stewardship principles to deter resistance, and financial constraints. This retrospective cohort study aimed to assess the magnitude and impact of delayed appropriate antibiotic therapy among patients hospitalized with septic arthritis (SA) in the U.S. from 2017 to 2019 using healthcare encounter data. Timely appropriate therapy was defined as the receipt of antibiotic(s) with in vitro activity against identified pathogens within two days of admission; all other patients were assumed to have received delayed appropriate therapy. Of the 517 patients admitted to hospital for SA who met all selection criteria, 26 (5.0%) received delayed appropriate therapy. In inverse-probability-treatment-weighting-adjusted analyses, the receipt of delayed appropriate therapy was associated with an additional 1.1 days of antibiotic therapy, 1.4 days in length of stay, and $3531 in hospital costs (all vs. timely appropriate therapy; all *p* ≤ 0.02). Timely appropriate therapy was associated with a twofold increased likelihood of antibiotic de-escalation during the SA admission.

## 1. Introduction 

Annual incidence of septic arthritis (SA) in the United States (US) and Western Europe is between two and ten cases per 100,000 persons, and is substantially higher among patients with rheumatoid arthritis—70 cases per 100,000 persons [1]. SA infections, which are most commonly caused by *Staphylococcus aureus*, are drivers of patient mortality, morbidity, and high healthcare costs [2,3]. In one observational study, over 80% of patients who presented to the emergency department (ED) for SA were hospitalized, with an average length of stay (LOS) of seven days and total annual charges of $30.6 to $36.9 million for ED care and $601 to $759 million for hospital care (USD) [3]. 

Among infectious diseases, delayed initiation of appropriate therapy is associated with increased risk of mortality, longer durations of antibiotic therapy and/or LOS in hospital, higher treatment costs, a decreased likelihood of being discharged home (vs. rehabilitation facilities or hospice), and long-term disability [2,4,5,6,7,8,9,10,11]. Microbiologic testing (including susceptibilities) for a suspected bacterial infection typically requires two to three days to complete [12]. Initial antibiotic therapy is therefore often empiric and based upon the infection and likely implicated pathogen(s). Early knowledge of causative pathogens thus reduces time to selecting appropriate therapy and potentially the magnitude of exposure to/duration of antibiotics [13]. 

To the best of our knowledge, the impact of delayed appropriate antibiotic therapy in SA is less well understood than in other infections. Accordingly, we utilized a large US hospital encounter database to examine the demographics, characteristics of admitting hospitals, clinical characteristics, infection details, treatment patterns, and outcomes among patients hospitalized for SA who received timely versus delayed antibiotic therapy. 

## 2. Materials and Methods

We used data from the Premier Healthcare Database (“Premier”) that spanned the period 1 January 2017 to 31 December 2019 (“study period”). Premier contains information on approximately 121 million inpatient visits and 897 million outpatient visits from >231 million patients seen at over 700 acute care hospitals, ambulatory surgery centers, and clinics [14]. The database includes patient demographics, hospital/facility characteristics, and data on admitting and discharge diagnoses (in International Classification of Diseases, Tenth Revision, Clinical Modification [ICD-10-CM] format); in addition it provides day-of-stay data on procedures (in International Classification of Diseases, Tenth Revision Procedure Coding System [ICD-10-PCS] format) and medications. Premier also includes financial data (defined as cost to institutions to render care). Laboratory test data—including specimen ID, test name, test day of service and time, and test results—are available for a subset of 370 hospitals [14]. Premier data are fully de-identified and compliant with the Health Insurance Portability and Accountability Act (HIPAA) of 1996 [14]. 

We identified all patients with at least one admission during the study period with a principal discharge diagnosis code of pyogenic (septic) arthritis (ICD-10-CM: M00.0XX, M00.1XX, M00.2XX, M00.8XX, M00.9) or direct infection of an unspecified joint in infectious and parasitic diseases classified elsewhere (M01X0) (see Supplementary Material for coding details). Principal diagnosis was used as it indicates the condition most responsible for admission. Among these patients, we focused on those who within two days of admission had: (1) ≥1 cultures from a relevant site (synovial fluid, blood, and related sites) for which microbiology data were available, and (2) receipt of ≥1 antibiotics (parenteral or oral preparations). For patients with multiple “qualifying” admissions, we selected the earliest admission (i.e., each patient contributed one admission to the analysis). Patients who died on the day of admission, and those transferred from other hospitals or discharged on the day of admission, were excluded. Consistent with prior studies [6,7,8,9,10,11], we excluded patients with evidence of other infection as it could not be ascertained from the database which treatment(s) were given for which infection; we also excluded those with evidence of conditions related to pregnancy or childbirth (additional factors not captured by the database may come into treatment considerations for such patients). For all remaining patients, the date of presentation to hospital was defined as the index date. Patients were followed from the index date until the earliest of either death, end of the study period, or 180 days post discharge (Figure S1). 

Timely appropriate antibiotic therapy was defined as the receipt of antibiotic(s) within two days of admission (i.e., date of initial presentation plus two days) that collectively provided coverage for identified causative pathogens. Delayed therapy was defined as the receipt of appropriate therapy any day thereafter while hospitalized. All antibiotics administered within two days of admission were classified as initial therapy.

### 2.1. Study Measures

Patient and hospital characteristics were assessed based on the available information from admission and during the 180 days prior (“pre-index”). Patient demographics included patient age, gender, race/ethnicity, and payer type; clinical characteristics included comorbidities (and the Charlson Comorbidity Index [CCI]), prior antibiotic use, prior hospitalizations and ED visits (all-cause and infection-related, with the latter defined as any encounter resulting in an infection diagnosis), and resource intensity [4,8,9,10,11]. Hospitals were classified based on geographic region, rural/urban setting, and teaching status. 

Organisms and corresponding susceptibility status were identified based on relevant cultures drawn within two days of admission. The proportion of patients with at least one ESKAPE organism (i.e., *Enterococcus faecium*, *S. aureus*, *Klebsiella pneumoniae*, *Acinetobacter baumannii*, *Pseudomonas aeruginosa*, *Enterobacter* spp.) was ascertained [15]. Antibiotic agents and treatment regimens received by patients during the index admission were also tabulated.

Antibiotic treatment patterns (e.g., escalation, de-escalation, unchanged) were evaluated among patients with ≥5 consecutive days of antibiotic treatment during admission. Escalation was defined as any increase in the quantity of antibiotics or spectrum score, or any change from oral to parenteral therapy (vs. the previous day); de-escalation, as any decrease in the quantity of antibiotics or spectrum score, or any change from parenteral to oral therapy. In cases where criteria for both escalation and de-escalation were met, a hierarchical approach was applied, as follows: (1) a change in route (oral vs. parenteral); (2) the number of unique antibiotics administered daily; (3) a reduction in spectrum score [16]. Only patients with ≥2 days of antibiotic therapy, LOS ≥ 3 days following the initial day of antibiotic exposure in hospital, and no evidence of death within five days following antibiotic initiation, were included in analyses of escalation/de-escalation [16]. Per Moehring et al. [16], escalation/de-escalation analyses were conducted from treatment initiation to day five of therapy or discharge (whichever occurred first), and to the last day of therapy/discharge, respectively.

Utilization included the duration of in-hospital antibiotic therapy (i.e., number of days of admission during which ≥1 antibiotics were administered), total antibiotic exposure (i.e., cumulative total days of antibiotic exposure during admission), and LOS. LOS was measured from day one (hospital admission) to the date of discharge. Costs included cost of antibiotics, other pharmacotherapies, room and board, medical care, and other costs of care; total in-hospital costs were the sum of these “component” costs. In all instances, these represented costs to the hospital to render care, and were presented in US dollar values for the year during the study period in which they were incurred. 

### 2.2. Statistical Analysis

For categorical variables, frequencies and percentages were reported; for continuous variables, means, medians, and ranges were reported. The statistical significance of differences between treatment groups was ascertained using Student’s t-tests for continuous measures, and chi-square tests for categorical measures.

Inverse probability treatment weighting (IPTW) was utilized to balance timely and delayed groups. Weights were estimated using the average treatment effect on the treated (ATT) methodology, in which the “treated” group (delayed appropriate therapy) were assigned a weight equal to one; corresponding weights for the “control” group (timely appropriate therapy) were estimated using propensity scores based on logistic regression models [17]. These scores were defined based on the outcome of interest (i.e., receipt of timely appropriate therapy) and were bound by zero and one. Demographic and clinical characteristics incorporated into this model included age group, gender, race/ethnicity, payer type, total number of hospital beds, teaching hospital status, obesity, CCI score, prior antibiotic use, markers of immunosuppression/general frailty, resource intensity, prior all-cause hospitalizations, and prior all-cause ED visits. Standardized differences were used to assess the degree to which IPTW methodology resulted in clinical equipoise between the groups (measures where standardized difference < 0.25 were considered balanced).

IPTW-adjusted utilization and cost outcomes were estimated using generalized linear models and a gamma distribution with a log link. As the presence of respiratory diseases remained unbalanced between the two groups following weighting, it was included in the models. To increase precision, we also included several covariates that were used in the IPTW process. Results from these analyses were presented as adjusted means, 95% confidence intervals (CI), and *p*-values.

All analyses were conducted using version 9.4 of SAS (Cary, NC, USA).

## 3. Results

### 3.1. Study Population

**Figure 1:**
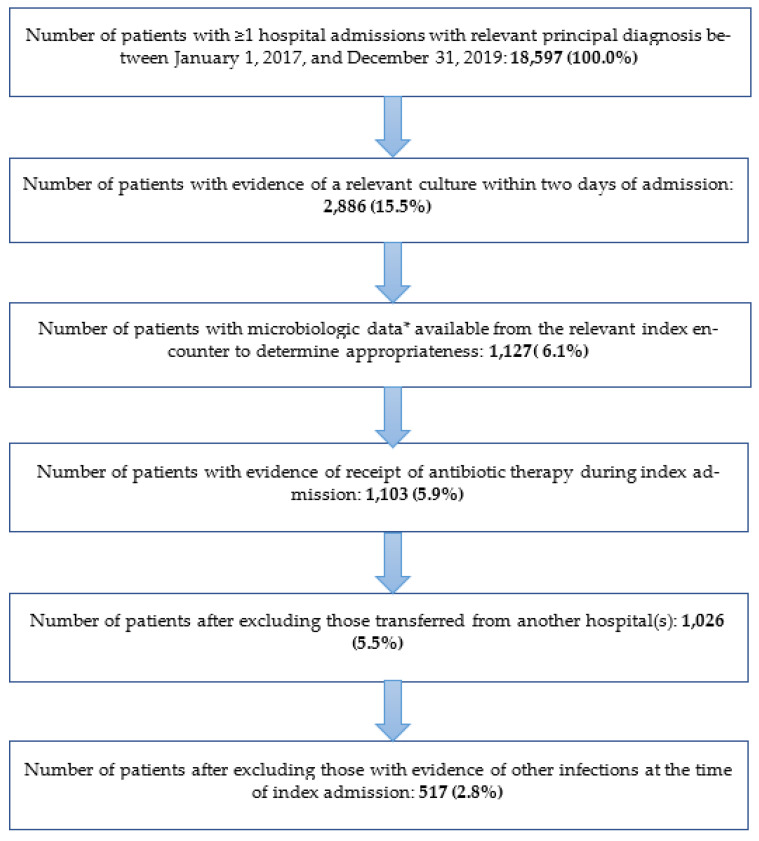
Patient attrition. Abbreviations: NA = not available. * Microbiologic data available in Premier included pathogens identified in culture draw and susceptibilities to various antibiotics, specimen source, and specific test utilized by the facility. Note: Zero (0) patients died on the day of index admission, were discharged on the same day as index admission, or had evidence of a condition related to pregnancy or childbirth during the time of index admission.

A total of 18,597 patients had at least one hospital admission for SA during the study period (1 January 2017 through 31 December 2019), of whom 1127 (6.1% of those with a qualifying admission) had microbiologic data available from relevant culture draws (Figure 1). Once all other selection criteria were applied, the study sample comprised 517 patients (2.8% of all admitted patients; 45.9% of those for whom microbiologic data were available). 

### 3.2. Organisms and Antibiotics Identified 

Thirty-two percent of culture draws were from synovial fluid, 10.0% from blood, and 57.8% from other sources (e.g., bone, tissue, other body fluid). Methicillin-susceptible *S. aureus* (MSSA) was the most commonly identified organism, and was present in 50.9% of the study sample; 12.6% had MRSA (Table 1). Other relatively commonly identified pathogens included *Streptococcus viridians* group (5.8%), *S. agalactiae* (3.3%), and *Pseudomonas aeruginosa* (2.7%). Eighteen percent had ESKAPE pathogens, most often MRSA. Most patients (83.4%) received vancomycin as part of their initial antibiotic regimen (either as monotherapy or as part of a multi-drug regimen) (Table S1). The most common initial regimens were ceftriaxone and vancomycin (11.4% of patients received this combination as their initial regimen), piperacillin/tazobactam and vancomycin (10.6%), vancomycin and cefazolin (9.9%), vancomycin monotherapy (10.6%), and cefepime and vancomycin (6.8%) (Table S2). 

Patients with timely appropriate therapy were about one-half as likely to have infections caused by ESKAPE pathogens versus patients with delayed appropriate therapy (16.5% vs. 38.5%; *p* = 0.004). For most patients (93.8%), SA was monomicrobial.

### 3.3. Patterns of Initial Therapy and Appropriateness Thereof

Initial antibiotic therapy was deemed appropriate for 491 patients (95.0% of the total study sample). Most patients (74.8%) who received timely appropriate therapy began it during day one of their hospital stay (Figure S2). 

Among patients who had ≥5 consecutive days of antibiotic treatment during admission (*n* = 464), 36.3% percent of patients in whom initial antibiotic therapy was deemed timely had de-escalated by the fifth day of treatment, versus 15.4% of patients in whom initial therapy was inappropriate (Table 2). Conversely, only 4.3% of patients who received timely appropriate therapy escalated their antibiotic therapy by day five (vs. 23.1% of patients in whom appropriate therapy was delayed). Between the date of therapy initiation and last day of treatment, 54.8% of patients who received timely appropriate therapy experienced de-escalation compared to 34.6% of patients who received delayed appropriate therapy. During the same time period, 9.6% of patients who received timely appropriate therapy experienced escalation, compared to 23.1% of patients who received delayed appropriate therapy.

### 3.4. Patient and Hospital Characteristics

Before weighting, mean age was similar between the two groups (~54 years of age), as was race (~75% White), geographic distributions of hospitals, and the proportion of hospitals that served urban populations (~90%) (Table 3). Patients who received timely appropriate therapy were more likely to be men (70.5% versus 57.7% of those who received delayed appropriate therapy; standardized difference = 0.27), and to have Medicare insurance coverage (33.6% versus 46.2%; 0.26); conversely, they had fewer chronic comorbidities (mean CCI 1.2 vs. 1.9 for patients in whom appropriate therapy was delayed; standardized difference = 0.40).

After IPTW, 412 patients (94.1%) were allocated to timely appropriate therapy, and 26 patients (5.9%) were allocated to delayed appropriate therapy. With few exceptions, demographic and clinical characteristics were balanced between the two groups following weighting (Table 4). Variables that remained imbalanced (i.e., standardized differences ≥ 25%) included diabetes without complications (27.8% of patients who received timely appropriate therapy vs. 15.4% of patients in whom such therapy was delayed; standardized difference = 0.31), hospitalization in the Northeast (30.0% of timely patients vs. 19.2% of delayed patients; 0.25), and hospitalization in a mid-size hospital (200–299 beds) (13.1% of timely patients vs. 3.8% of delayed patients; 0.34).

### 3.5. IPTW-Adjusted Utilization and Cost Outcomes

Patients who received timely appropriate therapy experienced significantly fewer mean days of in-hospital antibiotic therapy than patients who received delayed appropriate therapy (adjusted mean [95% CI]: 7.3 days [6.7–8.0] vs. 8.4 days [7.7–9.2]; *p* = 0.02) (Table 4). Additionally, the mean LOS was significantly shorter among patients who received delayed appropriate therapy (6.9 days [6.3–7.6] vs. 8.3 days [7.6–9.0]; *p* = 0.01). Mean antibiotic costs were significantly lower among patients who received timely appropriate therapy ($624 [$515–$756] vs. $1534 [$1286–$1829]; *p* < 0.001), as were mean costs of other pharmacotherapies ($1068 [$932–$1233] vs. $1639 [$1438–$1868]; *p* < 0.001) and medical care ($5861 [$5458–$6294] vs. $6521 [$6085–$6988]; *p* = 0.03). Consequently, timely appropriate therapy was associated with $3531 less in mean total in-hospital costs ($15,490 [$14,242–$16,846] vs. $19,021 [$17,528–$20,641]; *p* < 0.01).

## 4. Discussion

Among severe infections, timely initiation of appropriate antibiotic treatment is critical, as delays are associated with greater risk of morbidity and mortality, longer LOS, increased antibiotic exposure, and higher costs to provide care. Since the causative pathogen is typically unknown at presentation, initial therapy is often empiric, based on patient characteristics and knowledge of the implicated pathogen(s). Antibiotic stewardship further constrains clinicians in balancing comprehensive coverage against concerns of fostering resistance. Our findings indicate that receipt of timely appropriate antibiotic therapy for SA is associated with reduced exposure to antibiotics, shorter LOS, and an 18% reduction in costs to hospitals to render care.

Our findings are consistent with prior research indicating that the receipt of delayed appropriate antibiotic therapy among patients hospitalized with serious bacterial infections is associated with worse outcomes (all relative to the receipt of timely appropriate therapy) [4,15]. A high proportion of patients in our study sample received timely appropriate therapy, while similar studies in other disease areas suggest that delays in appropriate therapy occur more frequently (10–25%) [8,9,10,11,12,13,18]. This could be due to the high prevalence of *S. aureus* within our sample, which can be addressed with several antibiotics (including vancomycin, which also addresses MRSA, and was the most commonly used antibiotic agent in our study). 

Consistent with similar studies on SA, *S. aureus* was the most commonly implicated pathogen among our sample [2]. We also observed high-risk Gram-negative pathogens, including *P. aeruginosa*, *Escherichia coli*, *Enterobacter cloacae* complex, and yeasts (*Candida albicans, Candida parapsilosis*). These pathogens were more likely to be identified among patients in whom appropriate therapy was delayed, suggesting that clinicians may be less likely to provide appropriate coverage empirically for relatively atypical pathogens and instead require the results of microbiologic testing. This is consistent with other studies that note that these pathogens are associated with the receipt of delayed therapy [4,19]. Lodise and colleagues have noted that early knowledge of pathogens often results in a greater likelihood of timely and appropriate antibiotics [4]. If providers at hospitals within our sample utilized rapid microbiology testing for the 38.5% of patients in whom an ESKAPE pathogen was detected and appropriate therapy delayed, it may have resulted in a reduction of 14 days in hospital, 11 days of antibiotic therapy, and $35,310 (i.e., $3531 per patient) in total in-hospital costs. While traditional diagnostic methodologies such as synovial fluid culture typically require ≥48 h, molecular PCR panels can achieve results in a few hours, with accuracy reported equal or superior to traditional cultures; one study reported the sensitivity of synovial fluid and PCR to be 52% and 60%, respectively [20]. PCR has been found superior to culture in the identification of difficult-to-detect bacteria such as *Cutibacterium* spp. [20]. Use of rapid diagnostic technology provides results within a single provider shift, which allows for quicker and more efficient intervention with better follow-up such as minor dose and regimen adjustments (vs. waiting days for culture results to make adjustments). While the decision to purchase such technology is not always an easy one given resource-limited settings, such tools can often be used across infection types, thereby increasing not only their utility to clinicians in their selection process of antibiotic therapy and their detection of antimicrobial resistance markers but presumably also the cost-effectiveness/return on investment associated with such devices [21]. Without the availability of a rapid diagnostic tool, risk prediction tools also may help inform selection of empiric therapy (although further study is needed to determine the degree to which these tools accurately predict infection with various pathogens) [22].

This study classified treatment as timely if at least one agent within the initial regimen was appropriate for the identified pathogen(s), although this does not necessarily indicate that treatment was optimal; therapy regimens included extended-spectrum coverage (e.g., ceftriaxone and vancomycin for Gram-positive bacteria) or coverage of pathogens not identified (piperacillin/tazobactam and vancomycin for the treatment of Gram-negative bacteria). It is reasonable to assume that rapid pathogen identification afforded by rapid testing may reduce the use of overly broad antibiotic coverage (especially among monomicrobial infections), although clinicians are hesitant to discontinue double-coverage therapy in septic infections [23,24]. Among our sample, patients who received timely appropriate therapy were more likely to experience de-escalation—and less likely to experience escalation—compared to patients who received delayed appropriate therapy. Other studies have noted the key role that antimicrobial de-escalation (ADE) plays in stewardship and better clinical outcomes for patients with severe infection, including a decreased risk of mortality and lower rates of ICU admission [25,26,27]. Use of a rapid PCR panel may increase the speed at which ADE can be considered (where relevant). Even among patients in whom initial therapy is appropriate, earlier knowledge of causative pathogen(s) may allow for earlier initiation of a more suitable dosing regimen. Regardless of the benefits of ADE, providers may be hesitant to de-escalate therapy for fear that the infection may not improve (or even worsen). Despite the identification of MRSA among only one in eight patients, vancomycin was used in over three-quarters of all patients. Excessive empiric use of vancomycin contributes to resistance, with minimum inhibitory concentration (MIC) “creep” widely reported in the literature [28,29,30,31]. The ability for rapid pathogen identification may preclude the prescription of irrelevant antibiotics, thereby removing the need to consider de-escalation in some cases.

This study has several limitations. Capture of patient characteristics (e.g., overall levels of prior antibiotic exposure, comorbidities, infection history) within the database is limited to care rendered within hospitals that contribute to the database, resulting in the potential for misclassifying the timing of initial therapy and appropriateness as well as unmeasured confounding. This also led to an inability to characterize infections prior to presentation at hospital, including antibiotic use or culture findings prior to or after admission, limiting our ability to assess the association between the receipt of timely appropriate therapy and the overall duration of antibiotic therapy. On a related matter, outcomes also were restricted to those available in healthcare encounter data; data such as clinical efficacy evaluations from chart notes were unavailable and therefore could not be examined. Further study is needed to better understand how the timeliness of appropriate therapy impacts those evaluations. Premier lacks information on hospital policies that may influence prescription and treatment patterns. The degree to which these policies informed prescribing patterns observed in this study is unknown. Definitions of delayed appropriate therapy vary across existing literature from >24 h to >72 h following culture collection [32]. Premier antibiotic data are day-stamped but not time-stamped, which limited our ability to precisely capture the timing of the antibiotic receipt, resulting in possible misclassification of timely therapy. Without the availability of time-stamps, we used days since admission to classify timeliness, rather than assessing outcomes by actual testing turnaround time. While one systematic review reported that outcomes were similar across studies that defined delayed appropriate therapy as >48 h, >72 h, and at time of culture and susceptibility reporting (all relative to time of culture collection) [32], to the degree such misclassification occurred, it may at minimum have diluted the magnitude of delayed appropriate therapy. There also is no explicit linkage in the Premier database between the results of laboratory tests and specific diagnoses. Although we required a principal diagnosis of septic arthritis for inclusion in the study (which presumably was the reason that best explained the admission) along with a positive culture from a relevant site within two days of admission, and required review of all organisms and site among patients included in analyses by two members of the study team with substantial experience in SA, it is possible that some included culture draws may have been misclassified as relating to SA. However, without access to patients’ medical records, the degree to which misclassification occurred is unknowable. Finally, this study was limited to the microbiologic testing data available within the database; 85% of patients hospitalized for SA during the study period were excluded due to incomplete microbiologic data. This, and the knowledge that ours was a convenience sample, may limit the generalizability of findings.

## 5. Conclusions

In conclusion, when compared to timely appropriate therapy, those in whom such therapy was delayed had longer average LOS, longer duration of in-hospital antibiotic exposure, higher likelihood of escalation, lower likelihood of de-escalation, and higher healthcare costs. The ability to rapidly identify pathogens and susceptibilities is likely to reduce LOS, the duration of antibiotic exposure, and costs of care, while providing opportunities to enhance antimicrobial stewardship.

## Figures and Tables

**Table 1:** Frequency of Organisms Identified During Index Admission.

Pathogen	Study Sample (*n* = 517)
MSSA	263 (50.9%)
MRSA	65 (12.6%)
*Streptococcus viridans* group	30 (5.8%)
Methicillin-susceptible *S. epidermidis*	22 (4.3%)
*Serratia marcescens*	17 (3.3%)
*S. agalactiae*	17 (3.3%)
*Pseudomonas aeruginosa*	14 (2.7%)
Methicillin-resistant *S. epidermidis*	13 (2.5%)
*Enterococcus faecalis*	10 (1.9%)
*Escherichia coli*	8 (1.5%)
*S. pneumoniae*	7 (1.4%)
*Enterobacter cloacae* complex	6 (1.2%)
*S. dysgalactiae*	6 (1.2%)
*S. capitis*	5 (1.0%)
*S. hominis*	5 (1.0%)
Other *Staphylococcus* sp.	5 (1.0%)
Other strains of *K. pneumoniae* (non-ESBL)	4 (0.8%)
*S. gordonii*	4 (0.8%)
*S. pyogenes*	4 (0.8%)
*S. caprae*	3 (0.6%)
*S. lugdunensis*	3 (0.6%)
*K. oxytoca*	3 (0.6%)
*Neisseria gonorrhoeae*	3 (0.6%)
*Proteus mirabilis*	3 (0.6%)
Group C *Streptococcus.* sp.	3 (0.6%)
*Cutibacterium acnes*	3 (0.6%)
*Citrobacter* spp.	2 (0.4%)
*Morganella morganii*	2 (0.4%)
Vancomycin-resistant *E. faecium*	2 (0.4%)
*Haemophilus influenzae*	2 (0.4%)
*Candida albicans*	2 (0.4%)
*S. warneri*	1 (0.2%)
Other methicillin-resistant coagulase-negative *Staphylococcus* sp.	1 (0.2%)
*Achromobacter denitrificans*	1 (0.2%)
*A. xylosoxidans*	1 (0.2%)
ESBL *Klebsiella pneumoniae*	1 (0.2%)
*N. sicca*	1 (0.2%)
*Pasteurella multocida*	1 (0.2%)
*Raoultella ornithinolytica*	1 (0.2%)
*Providencia rettgeri*	1 (0.2%)
*Peptoniphilus* spp.	1 (0.2%)
Other *Enterococcus* sp.	1 (0.2%)
*S. cristatus*	1 (0.2%)
*Streptococcus* sp.	1 (0.2%)
Group B *Streptococcus* sp.	1 (0.2%)
*H. parainfluenzae*	1 (0.2%)
*C. parapsilosis*	1 (0.2%)
*S. zooepidemicus*	1 (0.2%)

Abbreviations: ESBL = Extended spectrum beta-lactamase producing; MRSA = methicillin-resistant *S. aureus*; MSSA = methicillin-susceptible *S. aureus*; sp = species.

**Table 2:** Patterns of antibiotic escalation and de-escalation, by timeliness of initial antibiotic therapy.

	Timely Appropriate Therapy (*n* = 438)	Delayed Appropriate Therapy (*n* = 26)	All Patients (*n* = 464) *
Until day five/discharge			
De-escalation	159 (36.3%)	4 (15.4%)	163 (35.1%)
Escalation	19 (4.3%)	6 (23.1%)	25 (5.4%)
No change	135 (30.8%)	6 (23.1%)	141 (30.4%)
Unknown	125 (28.5%)	10 (38.5%)	135 (29.1%)
Until last day of treatment/discharge			
De-escalation	240 (54.8%)	9 (34.6%)	249 (53.7%)
Escalation	42 (9.6%)	6 (23.1%)	48 (10.3%)
No change	154 (35.2%)	9 (34.6%)	163 (35.1%)
Unknown	2 (0.5%)	2 (7.7%)	4 (0.9%)

* Patients included in escalation/de-escalation analyses were required to have (1) ≥2 days of antibiotic therapy, (2) LOS of ≥3 days following in-hospital antibiotic therapy administration, and (3) no evidence of death within five days following initiation. As a result, overall *n* (*n* = 464) is lower than that of the overall hospital cohort prior to weighting (*n* = 517). Notes: All outcomes were measured from the end of day two alternatively to day five/discharge (whichever occurred first) and to the last day of treatment/discharge (whichever occurred first). Patients who did not receive at least five days of antibiotic therapy were excluded from these analyses. Patients who received ≥5 days of antibiotic therapy but did not have available data regarding escalation/de-escalation/unchanged were labelled as “Unknown”.

**Table 3:** Patient and hospital characteristics, by IPTW status and timing of appropriate therapy *.

	Unweighted Study Sample (*n* = 517)				Weighted Study Sample (*n* = 438)			
Variable	Timely (*n* = 491)	Delayed (*n* = 26)	Standardized Differences	*p*-Value	Timely (*n* = 412)	Delayed (*n* = 26)	Standardized Differences	*p*-Value
Demographics								
Mean (SD) age (years)	53.7 (18.4)	54.3 (18.9)	0.03	0.88	54.7 (5.0)	54.3 (18.9)	0.03	0.92
Male	346 (70.5%)	15 (57.7%)	0.27	0.17	243 (59.0%)	15 (57.7%)	0.03	0.93
Race/ethnicity								
White	365 (74.3%)	20 (76.9%)	0.06	0.55	317 (76.9%)	20 (76.9%)	0.00	0.79
Black	52 (10.6%)	3 (11.5%)	0.03		41 (10.0%)	3 (11.5%)	0.05	
Hispanic	26 (5.3%)	1 (3.8%)	0.07		18 (4.4%)	1 (3.8%)	0.03	
Asian	10 (2.0%)	1 (3.8%)	0.11		6 (1.4%)	1 (3.8%)	0.16	
Other	33 (6.7%)	0 (0.0%)	NA		26 (6.3%)	0 (0.0%)	NA	
Unknown/Missing	5 (1.0%)	1 (3.8%)	0.18		4 (1.0%)	1 (3.8%)	0.18	
Payer type								
Medicare	165 (33.6%)	12 (46.2%)	0.26	0.24	188 (45.7%)	12 (46.2%)	0.01	0.78
Medicaid	105 (21.4%)	7 (26.9%)	0.13		116 (28.1%)	7 (26.9%)	0.03	
Commercial	36 (7.3%)	0 (0.0%)	NA		16 (3.8%)	0 (0.0%)	NA	
Other	185 (37.7%)	7 (26.9%)	0.23		92 (22.4%)	7 (26.9%)	0.10	
Clinical Characteristics								
Comorbidities								
None identified	148 (30.1%)	7 (26.9%)	0.07	0.73	75 (18.3%)	7 (26.9%)	0.21	0.73
Asthma	75 (15.3%)	9 (34.6%)	0.46	0.01	116 (28.2%)	9 (34.6%)	0.14	0.01
Immunocompromised								
HIV/AIDS	2 (0.4%)	0 (0.0%)	NA	1	3 (0.7%)	0 (0.0%)	NA	1
Malignancies	19 (3.9%)	3 (11.5%)	0.29	0.09	27 (6.5%)	3 (11.5%)	0.18	0.09
Other	31 (6.3%)	3 (11.5%)	0.18	0.24	50 (12.1%)	3 (11.5%)	0.02	0.24
Total of above	49 (10.0%)	4 (15.4%)	0.16	0.33	76 (18.3%)	4 (15.4%)	0.08	0.33
Malnutrition/cachexia	26 (5.3%)	3 (11.5%)	0.23	0.17	37 (8.9%)	3 (11.5%)	0.09	0.17
Diabetes								
No complications	113 (23.0%)	4 (15.4%)	0.20	0.47	115 (27.8%)	4 (15.4%)	0.31	0.47
With complications	59 (12.0%)	5 (19.2%)	0.20	0.28	71 (17.2%)	5 (19.2%)	0.05	0.28
Total of above	129 (26.3%)	8 (30.8%)	0.10	0.61	133 (32.2%)	8 (30.8%)	0.03	0.61
Osteoarthritis	140 (28.5%)	5 (19.2%)	0.22	0.3	110 (26.8%)	5 (19.2%)	0.18	0.30
Obesity	94 (19.1%)	4 (15.4%)	0.10	0.8	60 (14.5%)	4 (15.4%)	0.02	0.80
Mean (SD) CCI Score	1.2 (1.8)	1.9 (1.8)	0.40	0.05	2.0 (0.5)	1.9 (1.8)	0.02	0.93
Markers of general frailty								
Corticosteroids	190 (38.7%)	10 (38.5%)	0.01	0.98	153 (37.2%)	10 (38.5%)	0.03	0.98
Vasoactives	98 (20.0%)	3 (11.5%)	0.23	0.45	80 (19.4%)	3 (11.5%)	0.22	0.45
Parenteral nutrition	0 (0.0%)	0 (0.0%)	NA	NA	0 (0.0%)	0 (0.0%)	NA	NA
Dialysis	5 (1.0%)	0 (0.0%)	NA	1.00	4 (0.9%)	0 (0.0%)	NA	1.00
Any of the above	245 (49.9%)	12 (46.2%)	0.08	0.71	196 (47.5%)	12 (46.2%)	0.03	0.71
Antibiotic use within 12 months of index	98 (20.0%)	8 (30.8%)	0.25	0.18	131 (31.7%)	8 (30.8%)	0.02	0.18
Antibiotic use within 6 months of index	34 (6.9%)	3 (11.5%)	0.16	0.42	54 (13.0%)	3 (11.5%)	0.05	0.42
Resource intensity >1 ^†^	183 (37.3%)	6 (23.1%)	0.31	0.14	5.52 (22.0%)	6 (23.1%)	0.03	0.14
Prior hospitalization within 6 months								
All-cause	78 (15.9%)	5 (19.2%)	0.09	0.65	81 (19.7%)	5 (19.2%)	0.01	0.65
Infection-related	40 (8.1%)	3 (11.5%)	0.11	0.47	49 (11.8%)	3 (11.5%)	0.01	0.47
Hospital Characteristics								
Geographic region								
Midwest	141 (28.7%)	6 (23.1%)	0.13	0.59	90 (21.9%)	6 (23.1%)	0.03	0.80
Northeast	123 (25.1%)	5 (19.2%)	0.14		124 (30.0%)	5 (19.2%)	0.25	
South	198 (40.3%)	14 (53.8%)	0.27		176 (42.7%)	14 (53.8%)	0.23	
West	29 (5.9%)	1 (3.8%)	0.10		22 (5.4%)	1 (3.8%)	0.07	
Number of beds								
<100	40 (8.1%)	0 (0.0%)	NA	0.26	16 (4.0%)	0 (0.0%)	NA	0.67
100–199	61 (12.4%)	3 (11.5%)	0.03		36 (8.9%)	3 (11.5%)	0.09	
200–299	73 (14.9%)	1 (3.8%)	0.39		54 (13.1%)	1 (3.8%)	0.34	
300–399	63 (12.8%)	3 (11.5%)	0.04		27 (6.5%)	3 (11.5%)	0.18	
400–499	61 (12.4%)	4 (15.4%)	0.09		38 (9.1%)	4 (15.4%)	0.19	
≥500	193 (39.3%)	15 (57.7%)	0.37		241 (58.4%)	15 (57.7%)	0.01	
Teaching hospital	277 (56.4%)	17 (65.4%)	0.19	0.37	271 (65.7%)	17 (65.4%)	0.01	0.98
Urban location	444 (90.4%)	25 (96.2%)	0.23	0.33	384 (93.2%)	25 (96.2%)	0.13	0.64

* Unless otherwise indicated, all values are number of patients (%). ^†^ Resource intensity defined as the ratio of a given patient’s total charges during the first two days in hospital to the total mean charges for the cohort during the same period. Abbreviations: CCI = Charlson Comorbidity Index score; HIV = human immunodeficiency virus; AIDS = acquired immunodeficiency syndrome; IQR = interquartile range; NA = not applicable; SD = standard deviation.

**Table 4:** Multivariable-adjusted utilization and cost outcomes.

Outcomes	Timely Appropriate Therapy (*n* = 412)	Delayed Therapy (*n* = 26)	*p*-Value
	Adjusted Mean (95% CI)		
Duration of in-hospital antibiotic therapy, days	7.3 (6.7–8.0)	8.4 (7.7–9.2)	0.02
Total in-hospital antibiotic exposure days, days	10.5 (9.7–11.5)	11.6 (10.6–12.6)	0.11
LOS, days	6.9 (6.3–7.6)	8.3 (7.6–9.0)	0.01
In-hospital cost, $			
Antibiotics	$624 ($515–$756)	$1534 ($1286–$1829)	<0.01
Other pharmacotherapies	$1068 ($932–$1,223)	$1639 ($1438–$1868)	<0.01
Medical care	$5861 ($5458–$6294)	$6521 ($6085–$6988)	0.03
Room and board	$7551 ($6818–$8362)	$7975 ($7223–$8805)	0.44
Other costs	$659 ($535–$812)	$587 ($481–$716)	0.44
Total in-hospital cost	$15,490 ($14,242–$16,846)	$19,021 ($17,528–$20,641)	<0.01

## Data Availability

Restrictions apply to the availability of these data. Data were extracted from Premier Healthcare Database owned by Premier Applied Sciences^®^, the Research Division of Premier Inc. Data may be requested with the permission of Premier Applied Sciences^®^, the Research Division of Premier Inc.

## Supplementary Materials

The following supporting information can be downloaded at: https://www.mdpi.com/article/10.3390/antibiotics11121732/s1. The following Supplementary Materials are included at the end of this document: Coding details; Figure S1. Diagram of the pre-index, index, and follow-up period; Figure S2. Receipt of appropriate antibiotic therapy by day of stay during index admission; Table S1. Frequency of selected antibiotics used as initial therapy during index admission; Table S2. Frequency of initial antibiotic regimens used during index admission.
