# Supplementary material for: Epidemiology and Economic Outcomes Associated with Timely versus Delayed Receipt of Appropriate Antibiotic Therapy among US Patients Hospitalized for Native Septic Arthritis: A Retrospective Cohort Study
Source: Antibiotics (Basel). 2022 Dec 1;11(12):1732. doi: 10.3390/antibiotics11121732 (PMC9774525; doi:10.3390/antibiotics11121732)
Supplement: Supplementary file 1 [file antibiotics-11-01732-s001.zip › antibiotics-2008426-supplementary.pdf]

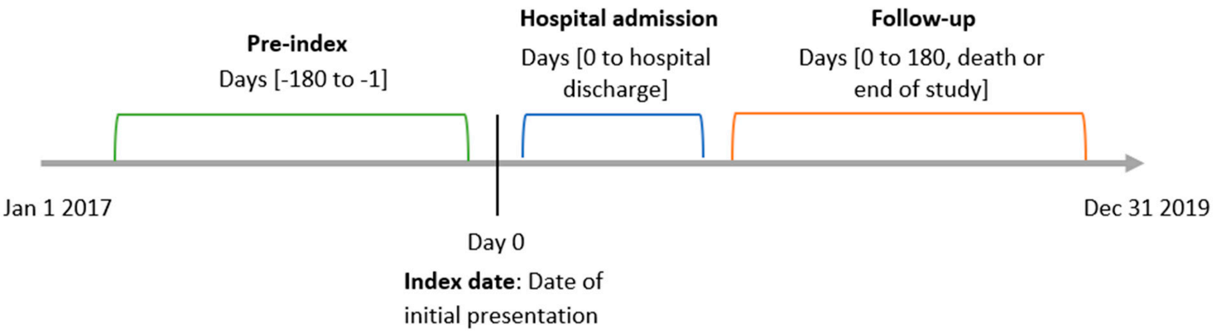

Figure S1. Diagram of the Pre-index, Index, and Follow-up Period.

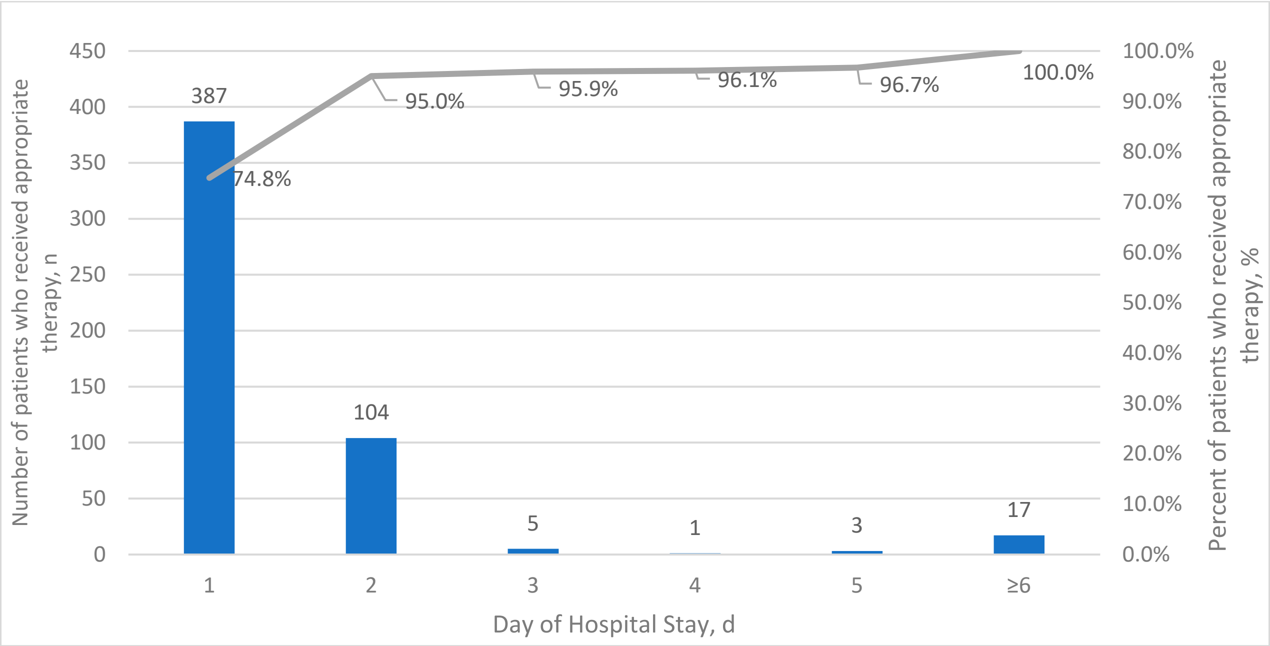

Figure S2. Receipt of Appropriate Antibiotic Therapy by Day of Stay during Index Admission.

Table S1. Frequency of Selected Antibiotics Used as Initial Therapy During Index Admission.

Variable	Study Sample (n = 517)
**Initial unique antibiotic regimen, *n* (%)**	
Vancomycin	431 (83.4%)
Cefazolin	154 (29.8%)
Ceftriaxone	149 (28.8%)
Piperacillin/Tazobactam	107 (20.7%)
Cefepime	79 (15.3%)
Clindamycin	43 (8.3%)
Daptomycin	21 (4.1%)
Meropenem	14 (2.7%)
Levofloxacin	12 (2.3%)
Nafcillin	12 (2.3%)
Tobramycin	11 (2.1%)
Ampicillin/Sulbactam	10 (1.9%)
Ceftazidime	10 (1.9%)
Gentamicin	10 (1.9%)
Ciprofloxacin	9 (1.7%)

Aztreonam	8 (1.5%)
Doxycycline	8 (1.5%)
Ertapenem	7 (1.4%)
Ampicillin	5 (1.0%)
Ceftaroline	5 (1.0%)
Metronidazole	5 (1.0%)
Linezolid	4 (0.8%)
Trimethoprim/Sulfamethoxazole (TMP-SMZ)	4 (0.8%)
Azithromycin	3 (0.6%)
Cefuroxime	2 (0.4%)
Cephalexin	2 (0.4%)
Oxacillin	2 (0.4%)
Amikacin	1 (0.2%)
Amoxicillin-Potassium Clavulanate	1 (0.2%)
Atovaquone	1 (0.2%)
Demeclocycline	1 (0.2%)
Minocycline	1 (0.2%)
Penicillin G Potassium	1 (0.2%)
Tigecycline	1 (0.2%)
Trimethoprim	1 (0.2%)
Cefadroxil	NA
Cefdinir	NA
Telavancin	NA

Abbreviations: NA = not available

Table S2. Frequency of Initial Antibiotic Regimens Used During Index Admission.

**Initial Antibiotic Regimen**	**Study Sample (*n* = 17)**
Ceftriaxone & vancomycin	59 (11.4%)
Piperacillin/tazobactam & vancomycin	55 (10.6%)
Vancomycin	55 (10.6%)
Cefazolin & vancomycin	51 (9.9%)
Cefepime & vancomycin	35 (6.8%)
Cefazolin	23 (4.4%)
Cefazolin & ceftriaxone & vancomycin	15 (2.9%)
Ceftriaxone & piperacillin/tazobactam & vancomycin	12 (2.3%)
Cefazolin & cefepime & vancomycin	8 (1.5%)
Cefazolin & piperacillin/tazobactam & vancomycin	8 (1.5%)
Cefepime & ceftriaxone & vancomycin	8 (1.5%)
Ceftriaxone	7 (1.4%)
Clindamycin & vancomycin	6 (1.2%)
Ampicillin/sulbactam	4 (0.8%)
Ceftriaxone & clindamycin & vancomycin	4 (0.8%)
Clindamycin	4 (0.8%)
Daptomycin	4 (0.8%)
Meropenem & vancomycin	4 (0.8%)
Aztreonam & vancomycin	3 (0.6%)
Cefazolin & clindamycin & vancomycin	3 (0.6%)
All others	149 (28.8%)
Total	517 (100%)

Abbreviations: ESBL = Extended spectrum beta-lactamase producing, MRSA = methicillin-resistant S. aureus; MSSA = methicillin-susceptible S. aureus; sp = species

Notes: “All others” comprised 125 unique initial regimens. This table includes multiple antibiotic exposures and drug regimen combinations over the length of admission among hospitalized patients.
